# Big data from small animals: integrating multi-level environmental data into the Dog Aging Project

**DOI:** 10.20506/rst.42.3349

**Published:** 2023-05

**Authors:** D. Xue, D. Collins, M. Kauffman, M. Dunbar, K. Crowder, S.M. Schwartz, Dog Aging Project Consortium, A. Ruple

**Affiliations:** 1 Institute for Public Health Genetics, University of Washington, 1959 NE Pacific Street, Seattle, WA 98195, United States of America; 2 Center for Studies in Demography and Ecology, University of Washington, 1901 Chelan Lane, Seattle, WA 98195, United States of America; 3 Department of Sociology, University of Washington, Seattle, WA 98195, United States of America; 4 Department of Epidemiology, University of Washington, 3980 15th Ave NE, Seattle, WA 98105, United States of America; 5 Department of Population Health Sciences, Virginia–Maryland College of Veterinary Medicine, Virginia Tech, 205 Duck Pond Drive, Blacksburg, VA 24061, United States of America

**Keywords:** Big data, Canine, Companion dog, Dog, Dog Aging Project, Environment, Geocoding, One Health, Open science, Canin, Chien, Chien de compagnie, Dog Aging Project, Environnement, Géocodage, Mégadonnées, Science ouverte, Une seule santé, Canino, Ciencia abierta, Dog Aging Project, Geocodificación, Macrodatos, Medio ambiente, Perro, Perro de compañía, Una sola salud

## Abstract

Environmental exposures can have large impacts on health outcomes. While many resources have been dedicated to understanding how humans are influenced by the environment, few efforts have been made to study the role of built and natural environmental features on animal health. The Dog Aging Project (DAP) is a longitudinal community science study of aging in companion dogs. Using a combination of owner-reported surveys and secondary sources linked through geocoded coordinates, DAP has captured home, yard and neighbourhood variables for over 40,000 dogs. The DAP environmental data set spans four domains: the physical and built environment; chemical environment and exposures; diet and exercise; and social environment and interactions. By combining biometric data, measures of cognitive function and behaviour, and medical records, DAP is attempting to use a big-data approach to transform the understanding of how the surrounding world affects the health of companion dogs. In this paper, the authors describe the data infrastructure developed to integrate and analyse multi-level environmental data that can be used to improve the understanding of canine co-morbidity and aging.

## Résumé

L’impact des expositions environnementales sur la santé est parfois considérable. Si diverses ressources ont été consacrées à décrire l’influence de l’environnement sur les humains, les efforts visant à étudier l’effet des paramètres environnementaux, tant naturels qu’anthropiques, sur la santé animale sont plus rares. Le Dog Aging Project (DAP) est une étude scientifique longitudinale à base communautaire portant sur le vieillissement du chien de compagnie. À partir d’observations notifiées par les propriétaires de chiens et de sources secondaires reliées par des coordonnées de géocodage, le DAP a réuni des variables sur le foyer d’habitation, l’environnement extérieur immédiat et le voisinage de plus de 40 000 chiens. Les séries de données environnementales du DAP couvrent quatre domaines : l’environnement physique et bâti ; l’environnement chimique et les expositions ; le régime alimentaire et la dépense physique ; et les interactions et l’environnement social. En combinant les données biométriques, les mesures du fonctionnement cognitif et comportemental et les dossiers médicaux, le DAP cherche à utiliser l’approche des mégadonnées pour transformer notre perception de la manière dont le monde qui nous entoure affecte la santé des chiens de compagnie. Les auteurs décrivent l’infrastructure des données mise au point pour intégrer et analyser des données environnementales multi-niveaux, afin de mieux comprendre les phénomènes de comorbidité et de vieillissement chez le chien.

## Resumen

La exposición a factores ambientales puede tener muchas e importantes repercusiones en los resultados sanitarios. Si bien se han dedicado cuantiosos recursos a aprehender la influencia del entorno en las personas, poco se ha hecho para estudiar el modo en que las características del medio, tanto natural como artificial, repercuten en la salud de los animales. El proyecto sobre Envejecimiento canino [Dog Aging Project: DAP] es un estudio longitudinal de ciencia ciudadana centrado en el envejecimiento de los perros de compañía. Combinando la información de encuestas realizadas a propietarios y de fuentes secundarias y vinculando los datos a coordenadas geográficas codificadas, el DAP ha permitido reunir información de variables ligadas al hogar, el jardín y el barrio de más de 40 000 perros. El conjunto de datos ambientales del DAP cubre cuatro grandes ámbitos: medio físico y urbanizado; condiciones químicas del entorno y exposición a sustancias químicas; régimen alimentario y ejercicio; y medio e interacciones sociales. Pasando por el uso combinado de datos biométricos, historias clínicas y mediciones de la función cognitiva y el comportamiento, el DAP apunta ahora a emplear técnicas de trabajo con macrodatos para hacer evolucionar nuestras ideas sobre la influencia del mundo que nos rodea en la salud de los perros de compañía. Los autores describen la infraestructura de datos establecida para integrar y analizar datos ambientales multiestratificados que nos ayuden a conocer mejor los procesos de comorbilidad y envejecimiento en el perro.

## Introduction

Advances in technology, social and economic upheaval, and the severe effects of climate change have altered the ways in which people interact with each other and the outside world, and will continue to shape social, built and chemical environments, resulting in immediate and long-term health consequences for humans and animals alike. Exposure to ambient air pollution is associated with higher risk for stroke, heart disease and respiratory infections [1, 2]. Built environmental features including green space and neighbourhood walkability have been shown to be positively related to cognitive function [3, 4]. Neighbourhood density and socio-economic status are associated with multiple facets of health and lifespan [5].

Companion dogs experience many of the same environmental exposures as their owners and develop many comparable health outcomes, yet little is known about how these exposures influence disease and lifespan in animals. Previous studies that have attempted to capture the effects of environment on disease in companion animals have been relatively small in sample size or restricted to specific geographic locations [6, 7].

The computational advances of the big-data era enhance the ability to compile, store and analyse environmental exposures at the population level. Using owner-reported surveys on household conditions and publicly available national environmental data sources, the Dog Aging Project (DAP) team has created an extensive household and neighbourhood environmental profile for more than 38,000 dogs currently enrolled in DAP. In this paper, the authors describe the infrastructure developed to compile data on a comprehensive set of longitudinal environmental factors that can be linked to canine co-morbidity and aging. Dog Aging Project data sets are available upon request (at https://dogagingproject.org/open_data_access).

## The Dog Aging Project

The Dog Aging Project is a large, national longitudinal study of aging in companion dogs [8]. The overarching goals of DAP are to understand the biological and environmental factors that shape aging and to discover how to maximise the healthy lifespan of dogs. In line with a One Health approach [9], a greater understanding of the relationship between the environment and dog aging can also have implications for human health. Companion dogs are an optimal species to study to better understand health and aging in humans since, in addition to living in the same environment as their owners, dogs have a robust healthcare system and develop many of the same diseases as humans, including cancer and dementia [10, 11]. It is also possible to learn more quickly about how the environment affects aging in dogs because they have shorter lifespans compared to humans. The Dog Aging Project collects survey data and electronic veterinary medical records on all dogs included in the project, as well as biospecimens from 10,000 dogs who form part of the study’s sampled cohorts. All of these additional data sources can be linked to an environmental profile based on the owner’s reported residential address. Further details on DAP have been previously described [8, 12].

The Dog Aging Project is built on a community science model. As of this publication, there are 41,517 dogs enrolled in DAP, and this number is constantly growing. Enrolment in DAP began in 2019 and all dogs are followed longitudinally. Thus, at the time of this paper, the dogs enrolled when the project was begun have four years of data associated with them. These enrolled dogs, often referred to as the DAP Pack, represent dogs of almost all breeds registered by the American Kennel Club as well as mixed breeds, of all ages and health conditions, located in all 50 states of the United States of America (USA). Furthermore, DAP data are readily available to investigators who are unaffiliated with the original study, upon request.

Environmental variables were carefully selected to fit this open-science model, with special attention being given to ensuring that all the environmental variables are publicly available and do not require further permissions to be shared. All of the data collected as part of the DAP data repository are available in a Google Cloud-based platform developed in collaboration with the Terra team at the Broad Institute of the Massachusetts Institute of Technology (MIT) and Harvard. The Cloud-based system allows seamless harmonisation, analysis and distribution of the hundreds of terabytes of DAP data. These data and further details, including codebooks, are available at https://dogagingproject.org/open_data_access.

## Multi-level environmental data in the Dog Aging Project

For each dog in the DAP Pack, the authors captured the environment at multiple levels, including the home environment, the yard or outside area where the dog is allowed to roam, the neighbourhood, and extra local areas where the dog regularly spends time. Across each of these levels, the authors focused on four environmental domains:

- the physical and built environment
- the chemical environment
- diet and exercise
- the social environment.

**Figure 1:**
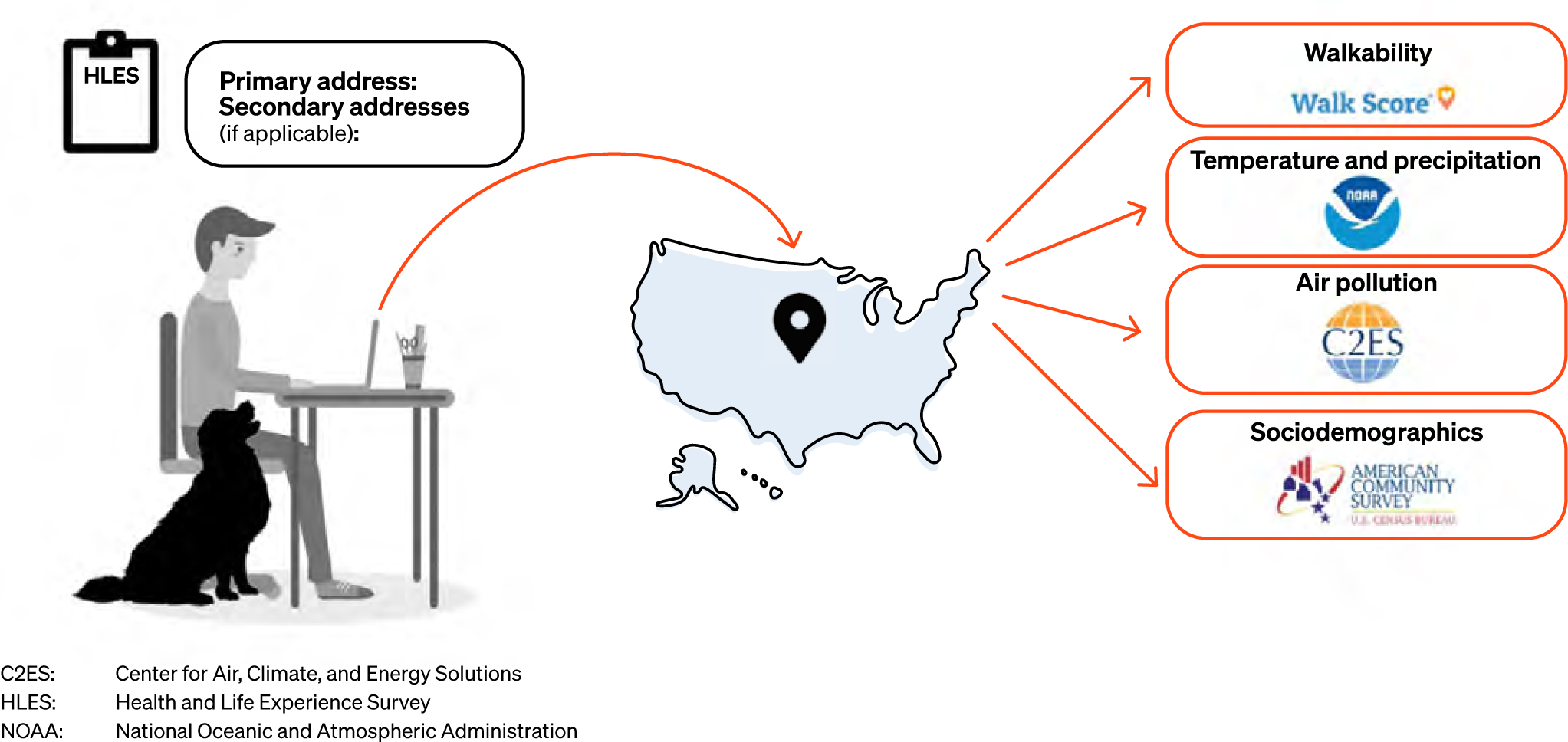
Conceptual figure of the Dog Aging Project data infrastructure Dog Aging Project (DAP) participants are asked to provide the primary and secondary addresses where their dog resides or spends time as part of the DAP Health and Life Experience Survey. These addresses are then geocoded to obtain spatial coordinates and Federal Information Processing Standards (FIPS) codes that can be linked to numerous publicly available secondary sources. Thus far, all geocoded addresses have been linked to data from the American Community Survey; Center for Air, Climate, and Energy Solutions; National Oceanic and Atmospheric Administration; and Walk Score. These environmental variables can be merged with data from other DAP surveys

The authors developed an owner-reported Health and Life Experience Survey (HLES) and geocoding system to link the owner-reported addresses to geographically based, publicly available secondary data sources, in order to collect information across environmental domains (Figure 1). As a result of the personally identifiable information captured in the survey and linked secondary sources, all dogs are assigned anonymised unique identifications (IDs) that are universal across all data sets.

## Owner-reported survey

The HLES contains eight sections:

- Dog demographics
- Dog behaviour
- Physical activity
- Environment
- Diet
- Medications and preventatives
- Health status
- Owner demographics.

Survey questions in the HLES were developed based on previous studies of dog health, including the Golden Retriever Lifetime Study [13] and Darwin’s Dogs (https://darwinsark.org), as well as human longitudinal studies [14, 15, 16]. During the design phase of the questionnaire, the authors met with small samples of dog owners within DAP to test and refine the survey questions, using test–retest strategies and conducting qualitative interviews to assess the interpretability and clarity of each question. The final HLES is delivered via Research Electronic Data Capture REDCap [17, 18] and made available for each participant through a password-protected portal. The final HLES is filled out at the time of enrolment and then annually thereafter.

Questions on the four environmental domains are dispersed across the eight HLES categories. The survey responses primarily describe the home and yard environment, with variables including ‘Number of people in the household’ or ‘Surface type of outdoor areas’. Examples of multi-level data across environmental domains are described in Table I.

## Geographic information system- and United States Census-based secondary data

In addition to the eight HLES sections, all owners are asked to provide a primary residential address and secondary addresses of extra local areas where their dogs spend time, if applicable. The study then uses these addresses as a key to unlock a rich array of secondary data, using a sophisticated geocoding procedure designed to ensure a very high proportion of matches between owner-reported residential addresses and geocoded addresses.

The authors used a three-tiered geocoding strategy. After testing several geocoding software packages on a subset of addresses, they determined that the Environmental Systems Research Institute’s ArcGIS Business Analyst geocoder [19] was the most successful, and subsequently designated it the primary geocoding method. Addresses that are not able to be matched using the primary method (3.1%) are then processed through the Smarty geocoder [20]. Finally, any addresses that remain unmatched are manually reviewed by the DAP team, using Google Maps and other readily available Web sources, such as Zillow (https://www.zillow.com) or Redfin (https://www.redfin.com). This three-tier method results in successful matches for 99.5% of all submitted primary addresses and 95.8% of secondary addresses. The addresses that the team is unable to match are often due to respondent typographical errors, entering a mailing address rather than a residential one (e.g. a PO Box number), or entering a newly built home.

Addresses that are precisely geocoded are then assigned spatial coordinates and United States (US) Census block-level Federal Information Processing Standards (FIPS) codes, which can then be linked to various publicly available data sources, including the American Community Survey (ACS) [21]; the Center for Air, Climate, and Energy Solutions (CACES) [22]; the National Oceanic and Atmospheric Administration (NOAA) Climate Divisional Database [23]; and Walk Score (https://www.walkscore.com). From these national databases, the DAP team obtained environmentally relevant variables and computed composite indices when appropriate.

Within the secondary databases, the authors focused on four domains:

- socio-demographic and economic factors
- air pollutants
- temperature and precipitation metrics
- walkability indicators.

**Figure 2:**
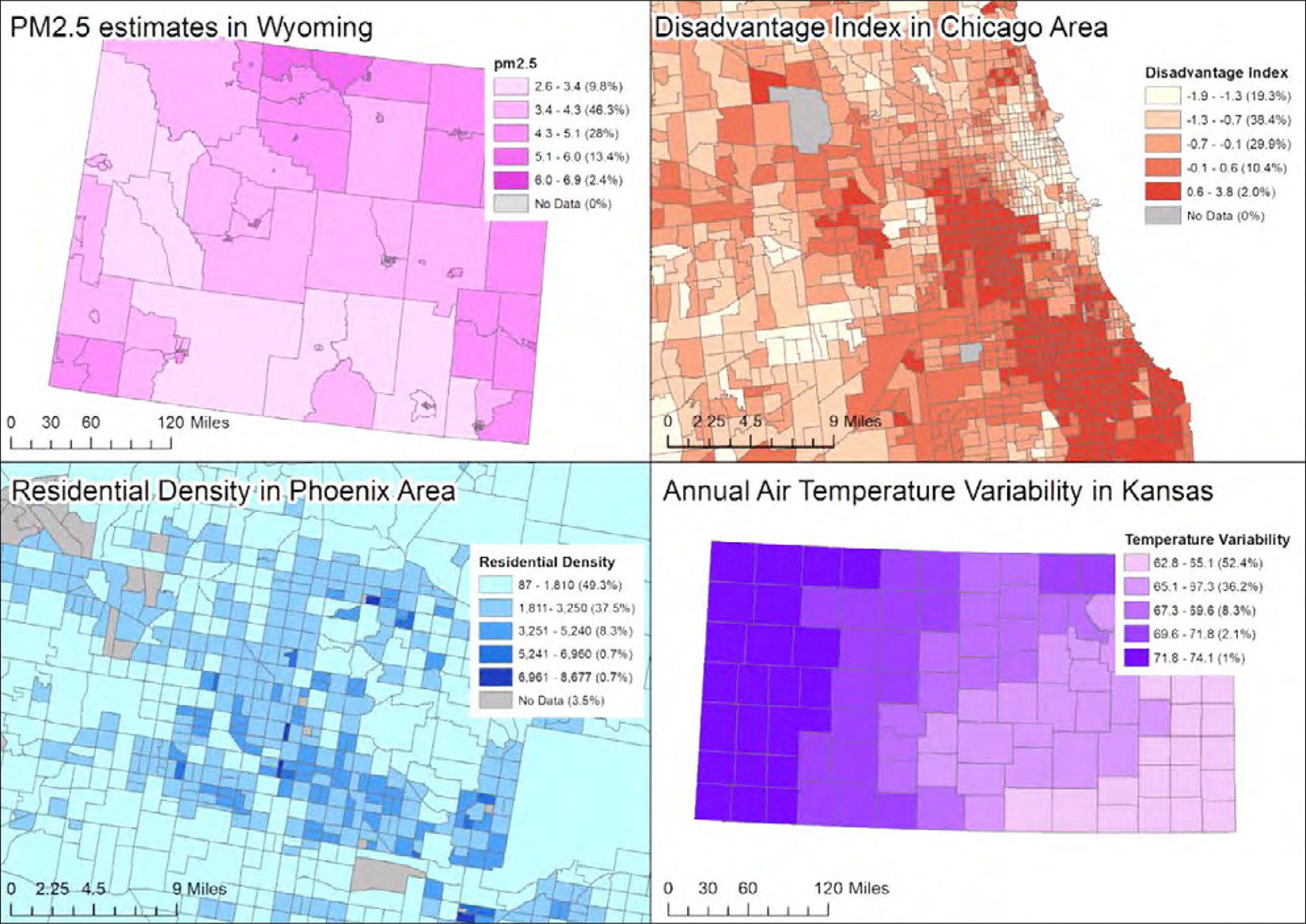
Environmental variability for selected regions and variables The variability of one variable from each environmental domain is depicted here, within a selected region. Each variable is split into five categories, with the proportion of Dog Aging Project participants residing in each respective category described in parentheses. Particulate matter_2.5_ (PM_2.5_) represents an estimate of long-term outdoor concentrations of fine particulate matter 2.5 micrometres or smaller. The disadvantage index is composed of five socio-economic variables: 1) Percentage of tract population below 125% of the poverty line; 2) Percentage of tract population aged 16–64 who are unemployed and not in the labour force; 3) Percentage of own children living in households with female householder, no male parent present; 4) Percentage of population 25 years or older with less than a bachelor’s degree; and 5) Percentage of households earning under US$ 100,000 (inflation-adjusted) in the past 12 months. Residential density is an estimate of the number of housing units in a census tract. Annual air temperature variability is a measure of difference between the average highest and lowest recorded temperature in the past year

They selected one representative variable from each domain as an example in Figure 2.

Socio-demographic and economic features are collected at the tract level from ACS (a census tract is the smallest territorial entity for which population data are available) Variables taken directly from the source include descriptive tract information (e.g. population estimate, area), demographics and economic indicators (e.g. median income, Gini index [24]). The authors then derived two composite variables based on the ACS variables: the disadvantage index and the stability index. Based on previous publications on neighbourhood effects and the relatedness of certain neighbourhood features, they computed the disadvantage index by averaging the z-scores (the number of standard deviations from the mean value of the reference population) of:

- the percentage of the population below 125% of the poverty line
- the percentage of those of working age who were unemployed or not in the labour force
- the percentage of children living in female-led households with no male parent present
- the percentage of population > 25 years with an educational level lower than a bachelor’s degree
- the percentage of households earning under US$ 100,000 in the last 12 months [25, 26, 27].

Similarly, based on collective efficacy literature, the team calculated the stability index by averaging the z-scores of the following variables:

- the percentage of the population who were in the same house one year ago
- the percentage of owner-occupied housing units
- the percentage of the population who were born in the USA [28].

Air pollution data are obtained directly from publicly available estimates developed by CACES, using version 1 empirical models, as described [29]. The most recent data include estimates of outdoor concentrations for six pollutants: ozone, carbon monoxide, sulphur dioxide, nitrogen dioxide, particulate matter 10 micrometres or smaller (PM_10_) and fine particulate matter 2.5 micrometres or smaller (PM_2.5_), each linked to geocoded addresses at the tract level.

Temperature and precipitation measures from NOAA are provided at the county level for two time periods. First, annual summaries contain temperature and precipitation averages for each month of every year since collection began. The DAP environmental data include annual summaries beginning in 2019. Second, ‘normals’ summaries are provided, which are long-term averages over 30-year periods with ten-year increments. The earliest set of ‘normals’ data used in DAP covers 1981–2010.

Finally, measures of neighbourhood walkability are derived from both Walk Score and ACS. Walk Score generates a walkability index based on walking routes to nearby amenities, using spatial coordinates (latitude/longitude). Using ACS, the authors included residential density variables at the Census tract level to compute a walkability score, based on prior findings that residential density is an appropriate proxy for objectively measured neighbourhood walkability [30].

## Longitudinal structure of environmental data

In addition to using unique, universal identifiers for each dog, which allows all variables to be merged across surveys and secondary data sources, the team has implemented several workflows to accommodate DAP’s longitudinal structure. It captures residential moves through an annual check-in survey. Owners are also able to update their residential address on their online DAP user profile at any time. Each address change is time-stamped, and secondary geocoded addresses are also updated annually.

### Data protection

The privacy and confidentiality of sensitive, personally identifiable information are a top priority in DAP. Addresses and geocoded coordinates are stored with extra security, and all survey results and linked secondary data are de-identified before being released for analysis. The original addresses used to link to secondary data sources will never be shared outside the environmental data management team. External investigators must complete a data-use agreement before they can access data that have been prepared for public release.

## Discussion

Big data will play a pivotal role in the future of both human and animal health. The DAP team has developed infrastructure to compile data on a comprehensive set of environmental risk factors that will allow it to gain new insights in aging and age-related disease in companion dogs. Here, the authors have laid out a blueprint for using individual addresses to uncover multi-level environmental data for each dog, and these data span social, physical and built environmental domains. Rather than relying only on owner-reported, individual-level information, environmental big data offer an opportunity to discover upstream, contextual determinants that can be targeted for population-level improvement of animal health.

The breadth of environmental factors covered through the owner-reported surveys and secondary sources can help researchers to measure the associations of environmental exposures. They can investigate direct associations between environmental variables and disease, such as measuring the association between exposure to particulate matter and cardiovascular disease. In conjunction with other survey responses and biospecimens collected as part of DAP, the DAP team can use the environmental data to conduct multi-level studies to understand how variation at the macro level modifies associations between individual-level factors.

For example, it is known that cognitive dysfunction is associated with chronological age [31]. However, by adding the environmental data, it is possible to investigate whether the level of association between age and cognitive dysfunction differs depending on the number of other animals or humans in the home environment, or whether neighbourhood greenspace density modifies the association between age and cognition. Furthermore, big-data projects in companion animals, such as DAP, can shift the paradigm in the understanding of the effects of gene–environment interactions throughout life course. These effects have been difficult to study in humans, as they require sample sizes of tens of thousands throughout a lifespan. Moreover, while individuals may be unwilling to provide their own genetic, epigenetic or metabolomic data due to privacy concerns, they may be less concerned about sharing this information for their dogs.

The initial curation of environmental variables was limited to information that is publicly accessible and informative for dogs nationwide, but the DAP data infrastructure is flexible for use in more geographically specific ancillary studies and is designed to seamlessly handle additional variables added in the future. Owing to the use of universal individual IDs that are consistent across data sets and time-stamped geocoding, new variables can be retroactively and prospectively added to the data structure as they become available. As an example, the DAP team is currently working on processing and incorporating Normalized Difference Vegetation Index (NDVI) values: indicators of density of vegetation or green space in a given area. Global NDVI values are available through the National Centers for Environmental Information and can be added retroactively to the DAP data set in future releases [32].

The project’s open-science model welcomes requests for ancillary studies, which provide opportunities for external investigators to add environmental variables that are only available in limited geographical areas to DAP’s existing scaffold. Some examples of geographically limited environmental phenomena that could impact dog health include fracking, wildfires or tick density. These data were not included in DAP’s original design, either because they were not widespread across the country or because geocoded data on these measures were not publicly available. However, such exposures can have ramifications for dog health that are worth investigating in ancillary studies [33, 34, 35, 36].

The project has some limitations. While DAP environmental data are expansive, covering both social and physical environmental features, the representation and socio-economic diversity of dog owners is limited (Figure 2). For example, the median income category of DAP participants is US$ 100,000 to $119,999, which is greater than the national average of $67,521 in 2020 [37]. Recruitment of the study population is volunteer-based and, as a result, the dogs enrolled in the study are those whose owners have Internet access and sufficient time to complete what can be a multi-hour assessment. Another potential selection bias is that owners who believe their dogs are unhealthy or living with serious medical conditions may be less likely to enrol them into a longitudinal study of aging. Lack of representation limits the generalisability of research findings. In the future, DAP will look towards other big-data initiatives, such as All of Us [38], that have implemented strategies to diversify participation.

The future of veterinary care, and health care as a whole, will increasingly rely on big-data methods. The Dog Aging Project goes one step further, beyond electronic veterinary medicine records and biometric data, by linking environmental big data to each participant record. While precision medicine is often thought of as treatments tailored to individual genomic patterns, and environmental or contextual determinants in the realm of public health, the integration of big data that span genomic, epigenomic and geospatial variation enables precision public health an overlap between two seemingly distinct fields. Owners go to great lengths to ensure the health of their dogs, but they might not be able to detect or control how environmental determinants, such as air pollution or precipitation, affect their dogs’ health. By simply providing an address, they enable DAP investigators to unearth potentially ‘unseen’ patterns, which can inform population-level interventions that enhance the effects of individual care.

## Figures and Tables

**Table I:** Variables collected from the Health and Life Experience Survey and secondary data sources Multi-level environmental data across four domains are collected as part of the Dog Aging Project. This table shows selected variants In each category. The full set of available variables can be found at https://dogagingproject.org/open_data_access. Variables in black are from the HLES; variables in orange are from GIS-linked sources

		Level			
		Home	Yard	Neighbourhood	Extra local
**Environmental domain**	**Physical and built environment**	Year built Stairs Pipes in home Fencing	Sun exposure Surface types	Sidewalks	Owner’s work environment
		Total size		Parks and green space Walkability score Average temperature Average precipitation	
	**Chemical environment and exposures**	Heating sources Cooking fuel Water source Water filtration Smoke exposure Flea and tick preventatives Lead paint Radon	Pesticide application and frequency Herbicide application and frequency	Traffic noise	Exposure to grooming products
				Nitrogen dioxide PM_2.5_ PM_10_ Ozone	
	**Diet and exercise**	No. of times fed per day Primary component of diet Secondary components of diet Treatment types and frequency Supplement types and frequency	Activity levels modified by local weather Activities with a lead or leash Activities without a lead or leash Bodies of water available for swim or play		
	**Social environment**	Number of animals Number of people Household income		Median household income	Accessibility of dog parks
				Stability index	Accessibility of ‘doggy day-care’
			Interactions with other animals and humans		

DAP: Dog Aging Project

GIS: Geographic information system

HLES: Health and Life Experience Survey

PM: Particulate matter
